# Prevention of Cyber Security with the Internet of Things Using Particle Swarm Optimization

**DOI:** 10.3390/s22166117

**Published:** 2022-08-16

**Authors:** Hassan A. Alterazi, Pravin R. Kshirsagar, Hariprasath Manoharan, Shitharth Selvarajan, Nawaf Alhebaishi, Gautam Srivastava, Jerry Chun-Wei Lin

**Affiliations:** 1 Department of Information Technology, Faculty of Computing and Information Technology, King Abdulaziz University, Jeddah 22254, Saudi Arabia; 2 Department of Artificial Intelligence, G. H Raisoni College of Engineering, Nagpur 440016, India; 3 Department of Electronics and Communication Engineering, Panimalar Engineering College, Poonamallee, Chennai 600123, India; 4 Department of Computer Science, Kebri Dehar University, Kebri Dehar 001, Ethiopia; 5 Department of Information Systems, Faculty of Computing and Information Technology, King Abdulaziz University, Jeddah 22254, Saudi Arabia; 6 Department of Mathematics and Computer Science, Brandon University, Brandon, MB R7A 6A9, Canada; 7 Research Center for Interneural Computing, China Medical University, Taichung 406040, Taiwan; 8 Department of Computer Science, Electrical Engineering and Mathematical Sciences, Western Norway University of Applied Sciences, 5063 Bergen, Norway

**Keywords:** artificial intelligence, cyber security threats, optimization techniques, particle swarm optimization, ant colony optimization, genetic algorithm

## Abstract

High security for physical items such as intelligent machinery and residential appliances is provided via the Internet of Things (IoT). The physical objects are given a distinct online address known as the Internet Protocol to communicate with the network’s external foreign entities through the Internet (IP). IoT devices are in danger of security issues due to the surge in hacker attacks during Internet data exchange. If such strong attacks are to create a reliable security system, attack detection is essential. Attacks and abnormalities such as user-to-root (U2R), denial-of-service, and data-type probing could have an impact on an IoT system. This article examines various performance-based AI models to predict attacks and problems with IoT devices with accuracy. Particle Swarm Optimization (PSO), genetic algorithms, and ant colony optimization were used to demonstrate the effectiveness of the suggested technique concerning four different parameters. The results of the proposed method employing PSO outperformed those of the existing systems by roughly 73 percent.

## 1. Introduction

As a result of increasing demand and expansion in the advanced network system of the Internet of Things (IoT), IoT concepts are becoming more complex every day [1]. The IoT is challenging to define because it has evolved and improved since it was initially introduced. Still, the best definition is a network of connected digital and analog computer devices with unique UIDs that can exchange data without a human being involved [2]. This is frequently considered a user interface for the centralized location system or application, typically a smartphone app that sends data or instructions to one or more edge IoT devices [3]. The peripheral can perform functions and transmit data to the primary computer system or application as needed, which a person can then access and use. IoT devices are vulnerable to Internet attacks because of various threat vectors, their uniqueness, and the absence of safety standards and guidelines. Hackers may use a range of cybersecurity risks against IoT devices, depending on the part of the network they target and the outcomes of the attack [4]. IoT-related cybersecurity research is therefore very active at the moment. Concerns regarding cyber security may be substantially helped by artificial intelligence [5]. Artificial intelligence may prove to be a helpful ally in the construction of defense against attackers. AI is capable of detecting and analyzing patterns for any anomaly [6,7]. This entails protecting IoT systems from hackers and using artificial intelligence to detect anomalous behaviour that might point to an assault. However, cybercriminals always have the upper hand [8] in the IoT scenario, since they only need to locate a hole, as opposed to cybersecurity experts who must secure several sites. As a result, cyber attackers increasingly turn to artificial intelligence (AI) to bypass sophisticated algorithms that can miss unusual behaviour [9,10]. IoT technology’s development has generated much interest in AI. Several AI optimization tools can now recognize potential dangers and activities in IoT cyber security applications as a result of this progress.

For several reasons, IoT applications are more susceptible to vulnerabilities than traditional computer systems. First of all, a variety of IoT systems are available, including devices, platforms, communication channels, and protocols. Second, rather than being created for Internet communication, IoT systems consist of “things” that are used to link physical systems. Third, IoT systems lack clearly defined limitations and undergo constant change due to the mobility of users and devices. Technical risks would also exist with IoT systems.

Last but not least, the restricted energy supply of IoT devices makes it challenging to deploy better security and solutions on linked devices [11,12,13]. Numerous nodes in an IoT ecosystem often govern lighting, heating, ventilation, air conditioning, and other services ranging from light detection, temperature, and noise to control systems. Through various networking protocols such as Bluetooth, Wi-Fi, RFID, etc., all sensors and control systems communicate with one another [14,15,16]. IoT gateways are utilized to connect these devices to the Internet. Each tier of the IoT ecosystem, which is made up of many levels of protocols, services, and technology, presents challenges for privacy protection. They can share data, limit the use of computer resources, and connect an enormous number of IoT nodes [17,18,19]. The rapid expansion of IoT-based devices will undoubtedly leave these networks more susceptible to challenges to privacy protection. Easily accessible IoT devices such as sensors have brought on numerous security issues in IoT networks. Because IoT devices have less processing power and appear to have a better signal than the present access point (AP) with the same service set identifier (SSID), the attacker has made all IoT devices vulnerable to connection to the software-enabled access point (SoftAP) [20,21,22]. This has made it possible for man-in-the-middle (MiTM) and eavesdropping attacks to compromise Internet communications. To develop IDSs and identify the hazards associated with IoT devices, such assault scenarios have been employed in IoT networks. The Internet of Things (IoT) concept is centered on the methods used to communicate with a real, physical world through the Internet [23,24].

Numerous nodes in an IoT ecosystem often govern lighting, heating, ventilation, air conditioning, and other services ranging from light detection, temperature, and noise to control systems. Through various networking protocols such as Bluetooth, Wi-Fi, RFID, etc., all sensors and control systems communicate with one another [25]. IoT gateways are utilized to connect these devices to the Internet. Each tier of the IoT ecosystem, which comprises many levels of protocols, services, and technology, presents challenges for privacy protection. They can share data, limit the use of computer resources, and connect an enormous number of IoT nodes [13]. The rapid expansion of IoT-based devices will undoubtedly leave these networks more susceptible to challenges to privacy protection. Easily accessible IoT devices such as sensors brought on numerous security issues in IoT networks. Because IoT devices have less processing power and appear to have a better signal than the present access point (AP) with the same service set identifier (SSID), the attacker has made all IoT devices vulnerable to connection to the software-enabled access point (SoftAP) [25]. This made it possible for man-in-the-middle (MiTM) and eavesdropping attacks to compromise Internet communications. To develop IDSs and identify the hazards associated with IoT devices, such assault scenarios have been employed in IoT networks. The Internet of Things (IoT) concept is centered on the methods used to communicate with a real, physical world through the Internet [26].

**Figure 1:**
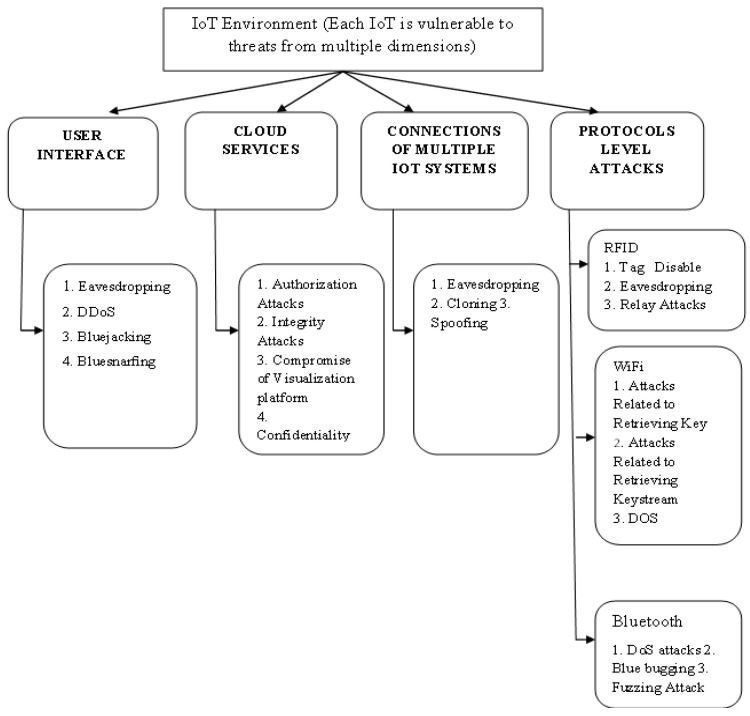
IoT environment threat dimensions.

For this reason, IoT settings feature several heterogeneous linkages and dependencies. Every connected ecosystem poses a cyber risk to every IoT system. IoT environments face threats from various dimensions, both real and virtual. Figure 1 deliberates the types of cyber security that are present in the IoT process, such as the interface from different users, variety of services from the cloud with multiple-system formation, and level of attacks [4]. In all the above-mentioned categories, a high level of attacks is present, and thus, these processes require high-security features at different dimensionalities. Even though multiple IoT systems are providing low attack features, the implementation of protocol-level features is much higher than that used by all individuals. Hence, a high-level feature needs to be provided to prevent any type of threat that enters the designed system.

### 1.1. Primary Literature Exploration

Ref. [1] presented the identification of a fake network node ‘on’ and ‘off’ assault in industrial IoT locations. It suggested that rogue nodes might target IoT networks while in an active or “on” state because of how they would turn on and off. In addition, the attacker node in the IoT network behaves normally, whether active or idle. A light-probe routing method was utilized to determine the confidence estimate of each surrounding node for an intrusion detection system. The authors of [2] developed a network traffic monitoring approach for all hypervisor-level virtual machines to protect the decentralized system. Using a binary bat approach with numerous targets was advised to properly determine the attributes. A warning was produced based on the outcomes of the random forest classification. A new signature for the assault was developed using the intrusion alarms from the various servers. The outcomes of both PSO and GSO are provided in terms of accuracy, where security boosting is highly enhanced by about 52 percent. However, this rise in accuracy does not guarantee protocol attack prevention and score, which is predicted in terms of the F-measure and is not measured [3]. The system’s evaluations were conducted using 22 benchmarking functions. The results show that the binary hybrid approach beats BGSA and BPSO.

Ref. [4] reported a hybrid fusion of the ABC and Adaboost algorithms. The ABC is utilized for the subset, and Adaboost characteristics are used to analyze, classify, and examine the device’s utility. It is recommended to use the ISCX1DS2012 and the NSL-KDD data sets to check the accuracy and detection rate. It has improved efficiency by comparing the proposed solution to an existing structure. Ref. [5] employed the PSO hybrid technique with rough sets to choose features well. The primary goal of the method being given is to increase classification accuracy while reducing the number of feature subsets. Across numerous datasets, the suggested strategy has proven effective as an attribute, instance, and class. One type of evolutionary algorithm has been introduced in double folds, where the presence of attacks is handled using deep learning models. This type of algorithmic integration is used at two levels to maximize the score of individual variables which provides more protection against service attacks [10]. Unfortunately, the test set only included a small number of assault types instead of a training set that would have evaluated participants’ ability to recognize them.

The limitations that are present using gateways [22] are that only corresponding nodes can access security features, whereas the remaining nodes remain in an idle mode of operation. Even some of the boundaries must be defined in transportation applications which are divided into separate layers, but all layers cannot be used at distinct periods [27,28,29,30,31]. In addition, high-end limitations are defined without any data-handling method, but more effectiveness can only be achieved if the data set is defined in a proper way [27,28,29,30,31,32,33]. In the case of intrusion detection and pathway management strategies [34,35,36,37,38,39], industrial operations are carried out, but basic limitations still exist in terms of application enhancement with two-directional security features.

### 1.2. Proposed Methodology

**Figure 2:**
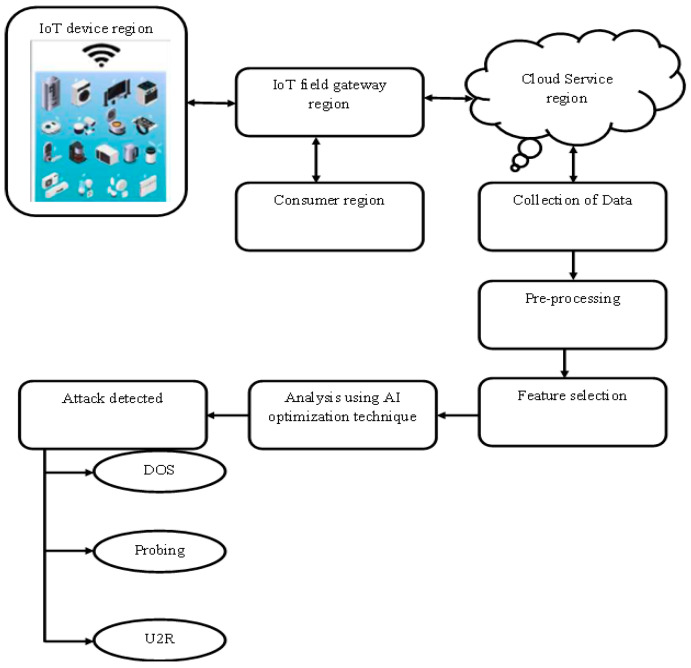
Block diagram for optimized hybrid artificial intelligence-based IoT-enabled cyber security system for a smart home.

In this article, we looked at a typical smart home application where a large number of IoT devices may be linked and controlled via an IoT gateway on the Azure host, as shown in Figure 2. The IoT device area, IoT field gateway area, Azure area, cloud gate area, and client region are the five sections that comprise the entire device. All of the IoT devices that have been installed in the smart home are located in the IoT Device zone [5,8,11]. The main control mechanism for the various parts of our smart home system is in the cloud region. Similar site sections are used to break up the Azure and Cloud Gateway zones. While Azure comprises multiple modules that monitor and manage all IoT devices, the Cloud Gateway area establishes links between the IoT Device Area and the Consumer Region. The client area also contains end-user interface gadgets (tablets, smartphones, etc.), which let a customer monitor the state of each IoT system as well as submit IoT applications to Azure components both online and offline [15]. Particle swarm optimization, ant optimization, and genetic algorithms are only a few of the optimization methods used in the approach’s main phases. The following subsections of the graphic detail each component of our home automation use case, and the visual contains data gathered from the NSL-KDD databases [17]. The blocks in Figure 2 represent multiple IoT devices that are installed in a particular region using wireless modules, where a gateway is directly connected for collecting secured data that is provided by a particular consumer. Once the data is transferred from the consumer, a separate encoded cloud monitoring system is then used for both pre-processing and collecting data at output units (Table 1). Further different features are selected by adding an artificial intelligence technique for recognizing the unformatted data in the entire system.

The aforementioned unformatted data is passed to the server station for checking the type of attack in the data. In case attacks are not detected, the data is taken in a particular way that is useful to individuals.

### 1.3. Objectives

One of the main objectives of this research is to design and build an IoT-based smart home. Smart home architecture is susceptible to IoT exposure to various cyberattacks, such as denial-of-service, data-type probing, and U2R attacks. To properly demonstrate the safety status of the IoT-based smart home system, it is required to identify and examine any safety risks. An optimization-based solution is offered to locate and protect the system in an abnormal state in this situation. Three optimization strategies have been applied to this problem.

## 2. System Model: Pre-Processing

The two datasets are the initial input data source for the experimental analysis. After that, the input data is prepared for sound and missing data removal [33]. The classifiers raised a great number of erroneous alerts as a result of the harsh characteristics. Preprocessing is essential as a result. Since some common qualities raise calculation time and memory requirements, classification procedures cannot be avoided. The NSL-KDD dataset classifies rough variables as follows [4],

(1) $$r_{s}=\left\{f_{s1}+f_{s2}+\ldots +f_{sn}\right\}$$

where *n* represents the dataset distinct characteristics.

As a result of the additional expense and redundancy, rough features do not include the usual features. The rough characteristics that have been modified [4] are shown as:

(2) $$r\dot{s}=\left\{f_{s1},f_{s2},f_{s3}\ldots \ldots \ldots f_{sp}\right\}$$

where *p* represents the best distinct characteristics.

After the elimination process, some weak traits are still present. After the dataset has been examined to ascertain its relative relevance, preprocessing is utilized to make the most of the feature collection. The study uses a variety of data preparation techniques for this aim, including data cleaning, normalization, integration, and description of each stage.

### 2.1. Data Cleaning and Normalization

Modifying data that has been duplicated, inaccurate, irrelevant, incomplete, or incorrectly framed is known as data cleansing. Data are not required for data analysis because it would be harder to make mistakes in findings. Information is removed by data cleansing in addition to being purged [35,36]. Incorrect data changes, data removal, and wiping of unnecessary information are all included in data cleaning. The primary goal was to exclude the information from the data sets that standardized the data analysis and made it easy to find the appropriate information for the investigation. Since there were already some incomplete or ambiguous data, it was necessary to alter the missing data to improve quality by removing bad information. When integrating and normalizing data, the MinMax normalization technique is crucial [37]. The highest feature value is changed to 1, and the lowest feature value is set to 0. All 0 and 1 values are converted to their binary equivalents. The normalization procedure [4] is described in Equation (3).

(3) $$R_{norm}=\frac{R_{i}-R_{min}}{R_{max}-R_{min}},$$

where *R_i_* represents data points, *R_min_* describes the value of the lowest data point, and *R_max_* denotes the value of the highest data point

All three variables determine the normalized value at two defined data points in the presence of structured data [32,33]. The data will still be questionable after the full normalization for unstructured information has been completed because of contaminated traffic data. The examination of assault prediction is made possible by collecting these traits from many complex systems [36].

### 2.2. Discretization and Integration of Data

The decentralization approach is used for discrete counterparts of periodic functions expressed in parameters [32]. When numerous discrete variables have been summed, it is known that the discretization technique alters the granularity category variable. The primary goal of the developed model is to reduce the amount considered for modelling applications [34]. The data integration focuses on the unique conceptual task of resolving multiple open challenges. Integration of data facilitated collaboration between internal and external users [35,36]. The collected information was added to the heterogeneous database, which already included reliable information for accessing customer files. The feature selection technique used to reduce the number of features is called Recursive Feature Elimination (RFE). According to the RFE, the feature numbers’ validity was unknown in advance, so the RFE helped choose and select the characteristics [37].

### 2.3. Feature Selection

When the data is taken from the RFE procedure, the feature values are automatically applied to the feature selection process, which aids in improving accuracy [38]. Unchecked functional values that are unnecessary, redundant, or irrelevant will no longer help categorize assaults. Therefore, key features are selected using feature selection methods to evaluate the search area’s accuracy. Based on relevance, the classifier eliminates the unimportant parts and chooses the top 10 features. Service, *Dst host srv count, Src byte, Dst byte, Dst host same src port rate, Count, Dst host diff srv rate, Srv error rate, Diff-srv rate*, and Protocol type are among the features. The strength of the exploration is increased by combining optimization approaches with exploration algorithms. Three optimization techniques are used to increase accuracy: genetic algorithms, ant colony optimization, and particle swarm optimization.

## 3. Analysis Using AI Optimization Procedure

This research evaluates the performance of three different classifiers using the data set mentioned above. To be more precise, we used the genetic algorithm, ant colony optimization, and particle swarm optimization.

### 3.1. Particle Swarm Optimization

**Figure 3:**
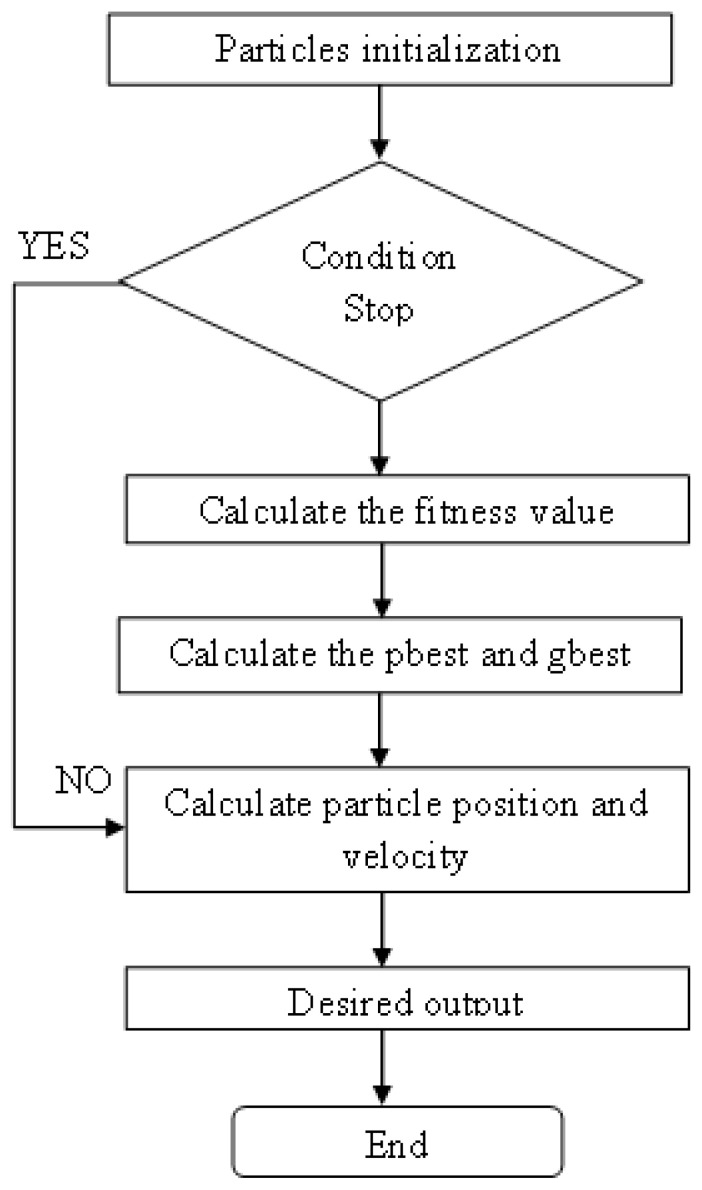
Flowchart of PSO algorithm.

The PSO algorithm, an SI global random search technique that imitates the migratory and swarming behaviour of feeding bugs, was developed by Kennedy and Eberhart. The traditional approach to each component of the swarm aggregation model is as follows: Every individual information must be protected, each information rate must be achieved in the immediate vicinity, and in the case of PSO, the information center must change independently of their destination. Particle swarm optimization (PSO) [34] identifies a particle in the search space for each optimization issue. The optimal function determines each particle’s fitness value, and its velocity determines its distance. Following the optimal particle, the particles will go through the subspace. The basic PSO algorithm’s flow diagram is shown in Figure 3 [39]. In the integration process, PSO is used with a determined analytical model for increasing the security of the data transfer process, and thus, different attacks that are present in the system are identified. Since PSO is chosen, the iteration values are set using a set of population matrices where each individual is given a specific set of fitness values that starts from 0.5 and ends at 1. The change in these two values provides a binary matrix that determines two individual best values that are denoted using variables p and g. The above-mentioned best values change according to each iteration between 10 to 100 in a step variation of 20. After determining the best value position of low-security elements, corresponding rapidity rates are measured as the output of PSO, where the speed of search space is increased with security measures. Further, the procedure of PSO does not require differentiable parameters, thus a providing great advantage of using the most optimal solutions in the entire process [37]. The optimum location that particle j has found is designated by the term Pbest [j], or the individual extremum. Gbest [j] stands for the global ideal point discovered by the complete particle swarm search. According to Equation (4), the particle positions and velocities are updated using the following random values for the subsequent generation.

(4) $$iter\left(i+1\right)=iter\left(i\right)\cdot h+\vartheta_{i}\cdot Rand_{i}\cdot \left(p_{best}\left(i\right)*g_{best}\left(i\right)-y_{i}\right)$$

(5) $$y_{i}\left(iter+1\right)=y_{i}\left(iter\right)+z_{i}\left(iter+1\right),$$

where *iter* describes the *i*th iteration of the current generation, $Rand_{i}$ indicates uniformly distributed random numbers between [0 and 1], $\vartheta_{i}$ represents the individual velocity value of each particle, and ‘*h*’ is the weight of inertia that dictates the particle speed before the current speed and functions as a balanced global search algorithm and local search capability.

The IPSO method’s accuracy falls as inertia weight accelerates convergence and improves the best solution. The suggested method converges too slowly but is more accurate because it has a smaller inertia weight. The inertia weight factor can be calculated to reduce the inaccuracy of the IPSO algorithm. Equation (4), the fundamental particle swarm algorithm [4], is rewritten as:

(6) $$\;z_{j}\left(k+1\right)=B_{1}+B_{2}+B_{3}$$

where

(7) $$B_{1}=z_{j}\left(k\right)$$

(8) $$\;\;\;B_{2}=\overset{n}{\underset{i=1}{\sum }}d_{i}Rand_{i}\cdot \left(pbest_{i}-y_{i}\left(iter\right)\right)$$

(9) $$B_{3}=\overset{n}{\underset{i=1}{\sum }}d_{i}Rand_{i}\cdot \left(gbest_{i}-y_{i}\left(iter\right)\right)$$

where $d_{i}$ indicates the dynamic speed rate of PSO search points.

The actual speed was substituted for the approaching rate for the existing B1, B2, and B3, which is the position most suited for accounting for the component effect on the current position [38].

### 3.2. Ant Colony Optimization

Pheromones are dispersed throughout the search area by the path in ACO, and the quantity of these pheromones indicates the trail’s strength. The ants prefer the direction of the track with the greatest amount of trail energy. One can suppose that the global system memory is the path’s most vital component [39,40]. Daemon activity is utilized to gather global data that is inaccessible to a single ant and use the data to assess whether more pheromones are required to aid with convergence. The algorithm is durable and messy in a dynamic environment via decentralized control. As an ACO, the system must decide whether to lose one ant or another to get through this uneasy decentralized structure. These crucial components work together to produce the shortest paths, which reflect the beginning phase, the middle condition of any system, and the outcomes of the ACO algorithm. A pheromone is released by,

(10) $$\omega_{ij}=\left(1-\mu \right)\omega_{ij}+\sum_{n=1}^{m}\omega_{ij}{}^{n}$$

where μ is the evaporation rate, m is the number of ants, and $\omega_{ij}{}^{n}$ is the quantity of pheromone laid by ant *n*.

### 3.3. Genetic Algorithm

The natural search algorithm serves as the foundation for the genetic algorithm. It uses the fitness survival tenet of Darwinian evolution theory. In a genetic algorithm, n members from each search space are explored by determining the energy rate by following four different steps, such as member support vector, reproduction stage, propagation factor, and pre-/post-processing stages, that minimize evolution procedures. Therefore, genetic algorithms mimic the evolution process. Every linage resembles an iteration, process, or succeeding linage when evolution is getting better and better [34]. Consequently, the objective function improves with each repetition. The fitness function of each of these chromosomes, sometimes called the evaluation or objective function, is encoded as a chromosome [39], also referred to as a genotype [40,41]. A chromosome’s fitness value impacts its capacity for resistance and procreation. Maximization is preferred based on the high fitness value, whereas minimizing is preferred based on the low fitness value [42]. In the case of the GA, two different representations are made after determining the type of data as genotype and phenotype. Whenever a genotype representation is made, the original data with a subset of the data type is then framed, but if the phenotype is used, then conversion is not processed as physical representations are made in the direct format. Moreover, both methods change concerning decision variables that are provided using search space depictions that contain separate chromosome values with variation in operational cases. Additionally, in GA, the random selection of data is not allowed, and thus, a sequential list must be arranged for processing data using mutation crossover.

In the case of swarm optimization, algorithms are combined, then parallel operations can be processed in some applications, and this is termed the binary swarm optimization process. The major applications in the combinational procedure are that different features are selected instead of standard ones, and thus, the accuracy of the binary model increases to a higher extent. Moreover, PSO and GA parameters are combined to predict the individual score of a particular application with a pre-processing technique. Once the data is processed, weighted combinations are chosen with the flip-pointing technique, thus preventing a high amount of data variations in the system. Further, the combination technique uses a controlling mechanism for preventing data attacks at a reduced cost of implementation.

## 4. Dataset

The most well-known IoT dataset is NSL-KDD. The NSL-KDD dataset comprises unique, redundancy-free sections that are copies of the original KDD Cup 75 dataset. There are 41 characteristics in the NSL-KDD dataset which are categorized as regular linkages or attack types. The KDD 75 dataset highlights several fundamental problems addressed in the NSL-KDD data collection [23,29]. There are a reasonable number of records and test sets in the NSL-KDD training. This is an advantage as it makes running the entire test set affordable instead of just picking a random, small portion. As a result, the evaluation results of different study efforts will be consistent and uniform. Three attacks by the NSLKDD, including DoS, U2R, and Sample Attack, are thoroughly described. The probe attack occurs throughout the network imaging procedure and is designed to abuse the data collected after the network information has been collected. Portsweep, Satan, Ipsweep, Mscan, Saint, and Nmap are examples of probing attacks that collect information from computers connected to the Internet [33].

After obtaining an ordinary account, U2R is given access to an account with root privileges. The attacks in U2R include buffer overflow, load module, Perl, SQLattack, Xterm, Rootkit, and Ps, to name a few [24]. A denial-of-service (DoS) attack occurs when a system cannot provide a service due to increased network traffic. Some DoS assaults that may be conducted against a target over the Internet are Neptune, Apache2, UDP Storm, Back, Land, Smurf, Teardrop, Worm, and Pod [35].

In Table 2, statistical values that are related to both training and testing phases are provided using the KDD data set, where abnormal values related to three distinct attacks are provided. In addition, the originally recovered normalized data is added to store the original data set attributes. Moreover, high data set values are trained in the proposed method, as compared to existing approaches where, for determining the presence of service attacks, more than 50,000 data are added. Similarly, the information that is passed in the training data set is completely trained in the entire process, and thus, normalized values are increased to 9823 per iteration cycle.

### Outcomes

To validate the performance measures, this work compares the hybrid optimization model’s predicted performance with those of three different optimization strategies. In this study, testing was conducted using NSL-KDD datasets. The suggested method uses the parameters listed to evaluate the results.

**Figure 4:**
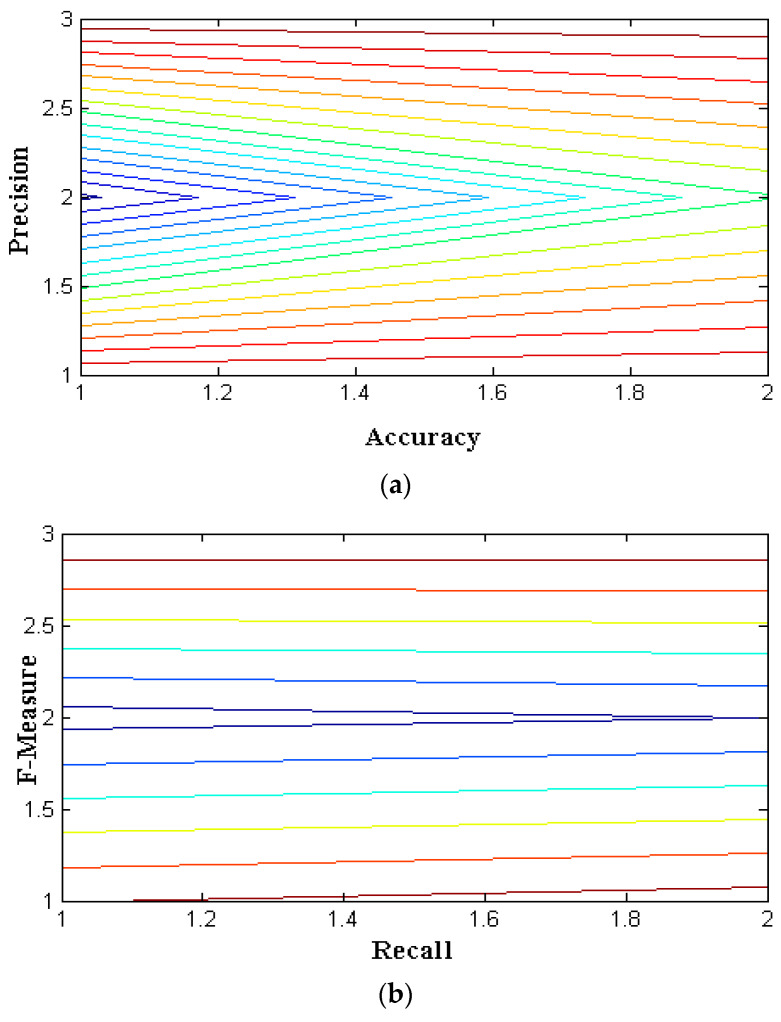
Performance metrics for GA with different attacks: (**a**) existing; (**b**) proposed.

**Figure 5:**
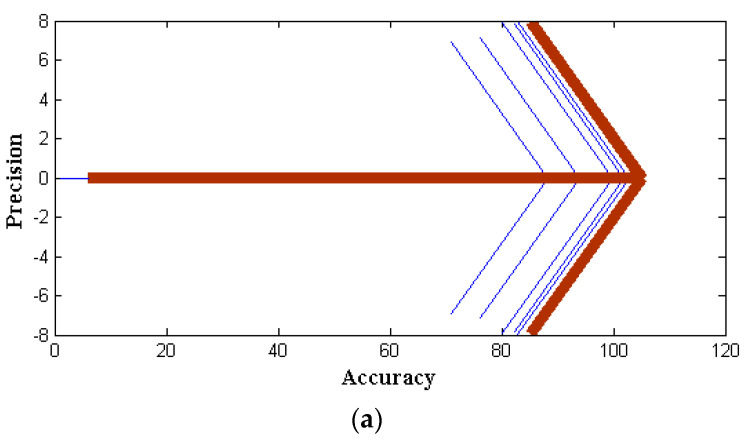

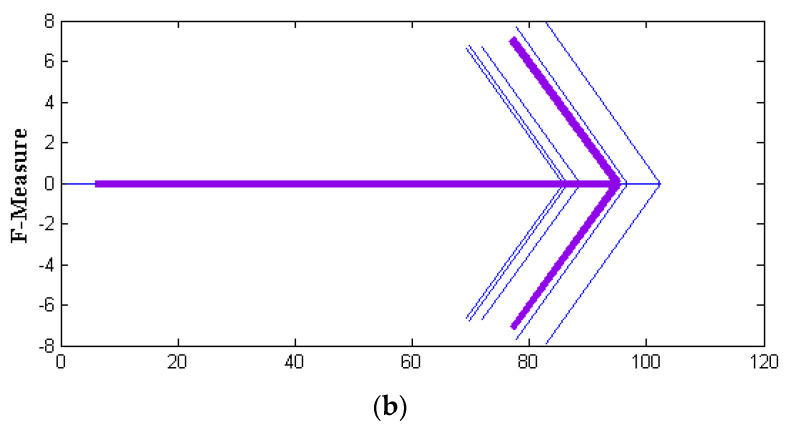
Performance metrics for ACO with different attacks: (**a**) existing; (**b**) proposed.

**Figure 6:**
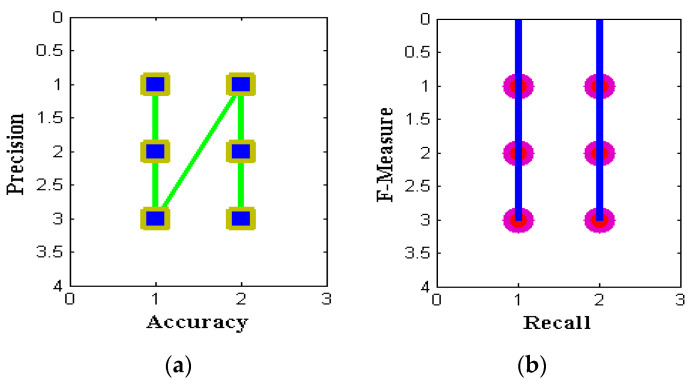
Performance metrics for PSO with different attacks: (**a**) existing; (**b**) proposed.

The outcomes of the proposed hybrid optimization approach are assessed using the efficiency attained for the binary classification of the NSL-KDD data set. The NSL-KDD dataset for multi-classification attacks is used to validate the results in Table 3 for attacks such as DoS, probing, and U2R. For each assault, the results’ precision, recall, accuracy, and F-measure are assessed. From Figure 4 and Table 3, it is observed that four parametric values that represent accuracy, precision, recall, and F-measure of three distinct algorithms are simulated. During this simulation process, two individual representations are made using subplot and contour programming code, and thus, colour values are provided to avoid complications. The accuracy and precision values of PSO provide optimal values as compared to the other two methods with nearly 99 percent values for service attacks. Similarly, the Fi rate of projected and existing methods is compared in Table 3, and corresponding values are plotted in Figure 5 and Figure 6. From the represented values in Figure 5 and Figure 6, it is very clear that the best values are achieved at low h values in the case of PSO.

**Figure 7:**
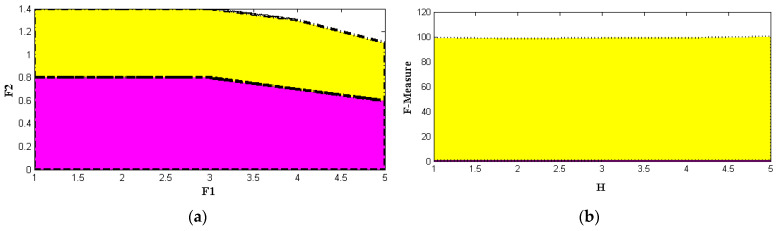
Parametric values (**a**) F1, F2, and (**b**) F-measure.

**Figure 8:**
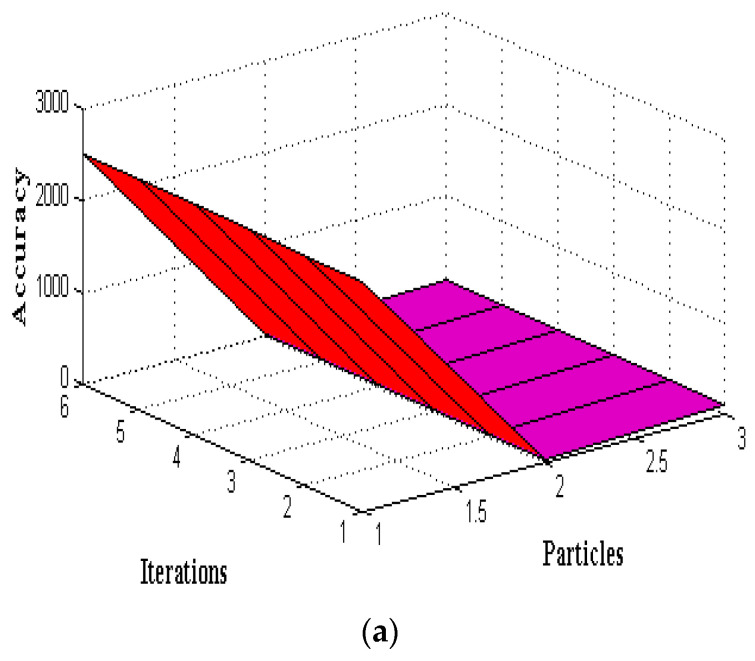

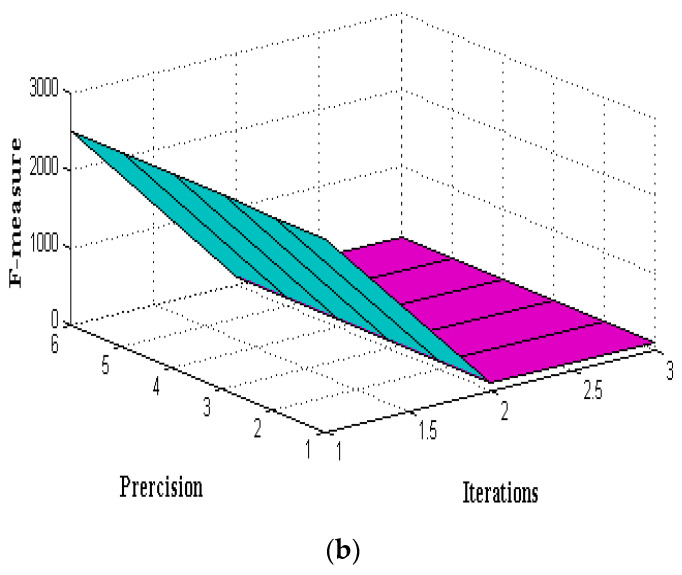
PSO algorithm using a fixed number of particles with increased iterations.

**Figure 9:**
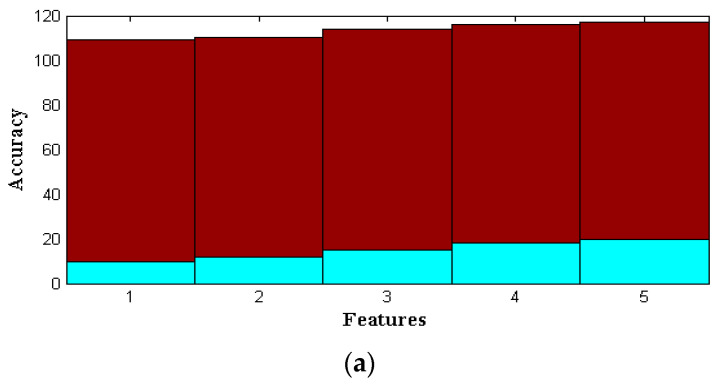

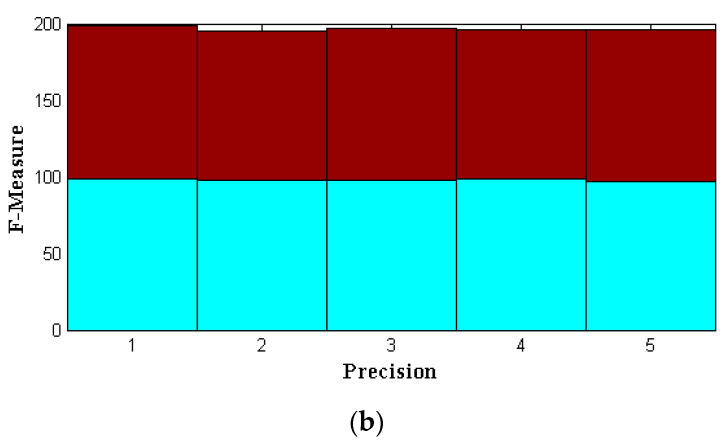
PSO algorithm with different feature sizes.

Even existing methods achieve 99% accuracy only after crossing 0.6 determination values at the last round. However, PSO achieves the same accuracy at the 0.5 iteration round even though its particles are higher, and thus, the increasing number of particles with high iteration values is plotted in Figure 7. The values that are represented in Table 4 are used for plotting three-dimensional illustrations where six iteration values from 25 to 30 are considered. These iteration values are changed concerning the same particle initialization, which is set at 2500. By using 2500 particles, the accuracy, predication score, and F-measure are increased concerning PSO as compared to GA and ACO by a high factor, rising to 97%. This increase provides the best feature extraction of 10 to 20, which is provided in Table 5 and plotted in Figure 8 and Figure 9. From Figure 9, it is pragmatic that accuracy and precision values are changed concerning different features, and thus, at 20 different feature extractions, PSO achieves 98% accurate service attack detection, whereas other feature extractions provide much lower service attack detection.

To assess the overall performance of the given strategy, we perform an analysis utilizing several PSO-selected attributes. The PSO parameters with the highest degree of precision are F1 = 0.6, F2 = 0.5, and h = 1.0. The test results for various parameters are shown in Table 4. We undertake several preliminary trials to determine the best empirical particle number and iteration combination. We find that 2500 particles and 29 iterations result in the final performance result shown in Table 5 and Figure 8. The same PSO configuration from Table 6 is used to examine this approach for various basic feature sets, including 10, 12, 15, 18, and 20 features. The outcomes are contrasted with those of a selection of 10 features shown in Figure 9.

If the network topology is rationalized to fifth-generation networks, then the process of handling IoT devices will be a much more challenging task as the design of a compatible IoT system is not built. In addition, IoT devices are highly vulnerable to the extraction of data, as, in the chosen route, many configuration flaws are present in the system. Even if the device is modernized, the system must not break all the violation rules that are allocated for a particular network configuration. However, the IoT is a free source that enables devices, where all the data is transmitted and stored in the system using a dynamic management strategy.

## 5. Conclusions

IoT devices are given a unique IP address that can be shared with the network’s external systems (i.e., users of a smart home). Since the number of assaults in the IoT ecosystem is increasing swiftly, safety issues with IoT devices are a serious concern. The data will be protected if the attacks by Internet hackers are stopped as they happen. Device capabilities vary between IoT tiers; as a result, different degrees of security-measure implementation have other elements and features. However, current methods are insufficient to detect and examine IoT malware. DoS attacks occur in IoT environments because of inadequate security monitoring and preventive tools. This paper uses hybrid particulate swarm optimization, ant optimization, and genetic optimization techniques to recognize attacks such as DoS, probe, and U2R. Even though the proposed method provides high-security features in IoT applications, some of the limitations are observed in case it is applied in practical cases. The foremost limitation of security constraints in IoT applications is that if attacks are processed in a large surface area, then no encrypted user can provide complete access control. Additionally, the execution environment which determines the level of security break in a particular data set is a major challenge, as some of the encrypted users with special keys transmit the data using deep-rooted software models that will force the external user to erase all necessary data in the entire storage system. However, all the above-mentioned limitations are solved in the proposed method using U2R procedures with a distinct protocol declaration.

As compared to other techniques, the particle swarm optimization method produces results with higher accuracy. The necessary plots prove that accuracy of the proposed method using PSO increases to 99% without any feature extraction procedures. On the contrary, in the case of feature extraction with 25,000 units, the proposed method provides 98% accuracy, which is much higher than the observed values in the existing method. Moreover, with iteration values from 25 to 30, PSO provides optimized results that increase the prediction and measurable score in the entire process. Therefore, the findings show that PSO outperformed both ant colony optimization and genetic algorithm optimization in terms of performance. In the future, the proposed work using PSO can be extended with multiple cloud computing platforms where the entire data set can be enhanced with high-security features. In addition, the extension is also possible by considering the separation of internal and external attacks where all users can transmit and receive multiple data using an artificial intelligence technique.

## Figures and Tables

**Table 1:** Comparison of the proposed technique with previous works.

Reference	Data Technique Used	Type of Algorithm	Objectives
[22]	Internet of Things	Artificial Intelligence	Cyber security operations with high network gateways
[27]	Layering procedure using Internet of Things	Artificial Intelligence	Compatibility of transportation applications with cyber security
[33]	-	Artificial Intelligence	Intelligent interactive devices for smart home applications with cyber security
[34]	Intrusion detection	Artificial Intelligence	Better service for cyber security operation and intelligent management
[39]	Pathway management	Artificial Intelligence	Increasing the secured operations for industrial applications
[40,41,42,43]	Deep generative model	Deep learning	Face recognition with a clone detection mechanism
Proposed	Internet of Things and cloud management	Artificial Intelligence	Building smart homes with enhanced cyber security features

**Table 2:** Statistical information about the NSL-KDD dataset.

KDD Dataset	Abnormal			Normal	Total
	DOS	Probing	U2R		
Training data	55,967	12,378	75	70,656	139,076
Test data	7590	3021	220	9823	20,654

**Table 3:** Performance metrics for different optimization techniques based on the attack detected.

Algorithm	Attacks	Accuracy (%)	Precision (%)	Recall (%)	F-Measure (%)
GA	DOS	98.90	98.90	94.90	96.89
	Probe	84.78	91.89	68.12	70.01
	U2R	99.90	99.78	99.67	99.21
ACO	DOS	98.89	97.95	95.87	98.45
	Probe	86.23	88.92	84.54	83.67
	U2R	99.87	99.05	82.76	88.94
PSO	DOS	99.50	99.93	99.54	99.65
	Probe	86.78	88.90	86.98	84.81
	U2R	99.98	99.67	99.01	98.34

**Table 4:** Algorithm parameters for the PSO using empirical data.

F1	F2	h	Accuracy
0.8	0.6	1.0	98.45
0.8	0.6	0.9	97.73
0.8	0.6	1.0	98.12
0.7	0.6	1.0	98.09
0.6	0.5	1.0	99.46

**Table 5:** PSO method results in utilizing a constant number of particles and increasing the number of iterations.

Particles	Iterations	Accuracy	Precision	F-Measure
2500	25	97.90	97.89	97.12
2500	26	98.06	97.03	97.56
2500	27	98.45	96.43	96.49
2500	28	98.23	97.63	98.62
2500	29	99.56	99.54	99.32
2500	30	97.96	97.87	97.51

**Table 6:** Observations of the PSO algorithm with different feature sizes.

Features	Accuracy	Precision	F-Measure
10	99.45	99.03	99.89
12	98.09	97.46	97.43
15	98.83	98.03	98.69
18	98.23	98.67	97.52
20	97.12	97.23	98.86

## Data Availability

The data presented in this study are available on request from the corresponding author.
